# Mapping the course to recovery: a prospective study on the anatomic distribution of early postoperative pain after total knee arthroplasty

**DOI:** 10.1186/s42836-023-00194-3

**Published:** 2023-08-03

**Authors:** Kevin L. Mekkawy, Bo Zhang, Alyssa Wenzel, Andrew B. Harris, Harpal S. Khanuja, Robert S. Sterling, Vishal Hegde, Julius K. Oni

**Affiliations:** Department of Orthopaedic Surgery, The Johns Hopkins University, 601 N. Caroline Street, Baltimore, MD 21287 USA

**Keywords:** Total knee arthroplasty, Postoperative pain, Pain mapping, Pain location

## Abstract

**Introduction:**

Early postoperative pain following total knee arthroplasty significantly impacts outcomes and patient satisfaction. However, the characteristics and sources of early pain after total knee arthroplasty remain unclear. Therefore, the purpose of this study was to determine the anatomic distribution and course of postoperative pain in the acute and subacute period following total knee arthroplasty.

**Methods:**

A prospective observational study of primary, elective unilateral total knee arthroplasty cases was conducted at our academic tertiary care medical center from January 2021 to September 2021. Preoperative variables were extracted from institutional electronic medical records. Postoperatively, patients utilized a knee pain map to identify the two locations with the most significant pain and rated it using the visual analog scale (VAS). The data were collected on day 0, at 2 weeks, 2 months, and 6 months after operation.

**Results:**

This study included 112 patients, with 6% of patients having no pain at postoperative day 0, 22% at 2 weeks, 46% at 2 months, and 86% at 6 months after operation. In those who reported pain, the VAS score (mean ± standard deviation) was 5.8 ± 2.4 on postoperative day 0 and decreased at each follow-up time point (5.4 ± 2.3 at 2 weeks, 3.9 ± 2.2 at 2 months, and 3.8 ± 2.7 at 6 months). The majority of patients were able to identify distinct loci of pain. The most common early pain loci were patellae, thigh, and medial joint line, and this distribution dissipated by 6 months.

**Conclusion:**

At 2 postoperative weeks, pain was primarily at the medial joint, and at 6 months postoperatively, pain was more likely to be at the lateral joint. No relationship was found between pain at six months and pain scores or location at postoperative day 0 or 2 weeks. Understanding the distribution and progression of knee pain following total knee arthroplasty may benefit patient education and targeted interventions.

**Level of Evidence:**

Level II, prospective observational study

**Supplementary Information:**

The online version contains supplementary material available at 10.1186/s42836-023-00194-3.

## Introduction

Total knee arthroplasty (TKA) is the gold standard for treating end-stage osteoarthritis of the knee, with annual 3 million cases projected in the USA by 2030 [1]. While most patients experience improvement in pain, function, and overall quality of life, there remains significant room for the improvement in patient satisfaction and pain relief. About 72%–86% of patients reportedly were satisfied with pain relief and 75%–89% reported overall satisfaction after TKA [2, 3]. Although surgical complications, such as infection, or mechanical problems such as aseptic loosening or malalignment can cause postoperative pain, early postoperative pain following TKA is often not attributable to a specific mechanical or infectious complication [4].

In general, pain after TKA tends to be most pronounced in the subacute postoperative period, with approximately half of patients experiencing severe pain in the first two weeks following TKA [4]. Within 3 months postoperatively, two-thirds of patients reported pain with alterations in quality of life and sleep [5]. Thus, post-TKA pain represents a significant area for potential interventions to maximize desired TKA outcomes and satisfaction. Patients who suffer from protracted post-TKA pain without an attributable cause present a substantial dilemma, and reoperation is not recommended [6, 7]. Thus, in the immediate postoperative period when unexplained pain is more common, pain management is crucial to achieving desired outcomes, and this can exert a substantial impact on hospital course, rehabilitation, and the development of chronic pain [5, 8–10]. To better understand the cause of unexplained post-TKA pain, it is necessary to characterize the course and location of pain. Although existing literature describes anterior knee pain to be most prevalent, to our knowledge, no reports described a specific foci or distribution of pain around the knee in TKA patients [2, 11, 12].

This prospective observational study aimed to establish the most common pain sites and distribution patterns on the day of surgery, and 2 weeks, 2 and 6 months after TKA. We hypothesized that pain scores would decrease at each time point, and that those with more severe pain in the immediate postoperative period would have greater pain scores throughout the study.

## Materials and methods

This study was approved by our institutional review board, with a registration number of IRB00131215.

We conducted a prospective observational study in subjects receiving primary elective unilateral TKA performed at our academic tertiary care medical center by four adult reconstruction surgeons from January 2021 to September 2021. All cases of unicompartmental knee arthroplasty, revision arthroplasty, simultaneous bilateral TKA, and TKA for indications other than primary osteoarthritis were excluded from this study. Power analysis was performed based on prior studies by utilizing a knee pain map assessment, in which we determined that 100 cases of TKA sufficed to reach statistical power [13]. All surgeons used a medial parapatellar approach with an anterior midline incision. Anesthetic interventions included spinal anesthesia with sedation when available, periarticular injection of 0.5% ropivacaine and ketorolac, and a postoperative oral pain regimen. In patients who were not amenable to spinal anesthesia, general anesthesia was performed. No nerve blocks were used.

### Data collection

**Fig. 1:**
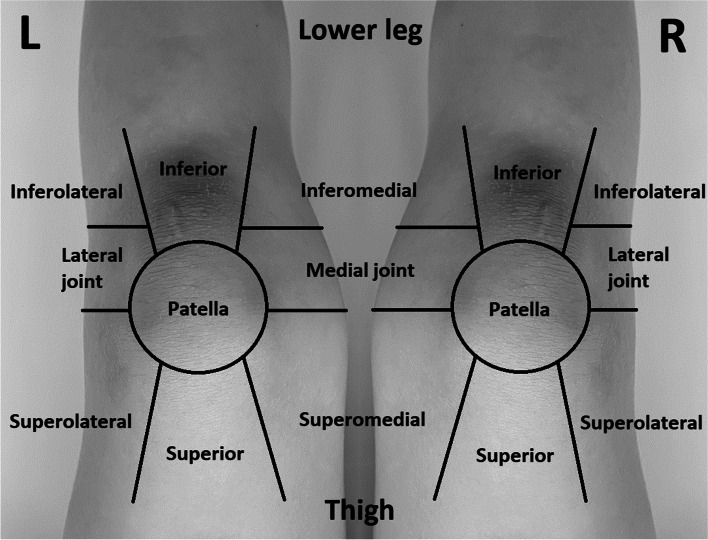
Knee pain map utilized by patients to identify location(s) of their most significant pain

Preoperative variables were extracted from institutional electronic medical records. These variables included patient age, sex, body mass index (BMI), laterality of the procedure, and medical comorbidities. On postoperative day 0 (POD 0), patients were asked to utilize the provided knee pain map **(**Fig. 1**)** to identify/report the two most severe locations of their pain, and to rate the pain, by utilizing the visual analog scale (VAS), with scores ranging from 0 to 10. The locations of pain included inferior, inferomedial, inferolateral, patellar regions, medial joint, lateral joint, superior, superomedial, superolateral, posterior areas, or were diffuse, or had no pain. The knee pain map is based on a clinical image of the knee from the point of view of the patient. It was designed on the basis of prior studies on native knee pain in osteoarthritis patients. These studies concluded that an anatomically-based knee pain map allowed patients to successfully pinpoint knee pain with an excellent inter-rater reliability in terms of pattern and location [14]. In addition, this method was shown to have very good inter-rater and intra-rater reliability, and work well when used by the patient [15, 16]. Due to COVID-19-related restraints at the beginning of our study, in-office follow-up was restricted for certain periods of time. Thus, when necessary, patients were subsequently surveyed via telephone 2 weeks, 2 and 6 months after operation. The location and severity of their knee pain were assessed in the same fashion as the patients who localized pain using the aforementioned knee pain map.

### Statistical methods

Categorical data were presented as frequencies with percentages and continuous variables were expressed as means ± standard deviations. Mean pain scores were calculated for each pain location at each follow-up time point. To account for patients being asked the same questions about location and severity of pain at various follow-up time points, a mixed effects model was created. The pain score and location with the greatest severity were then used in the model. Analyses were conducted by using ANOVA, including calculation of effect size. Post-hoc analyses were also performed using Tukey’s Honest Significant Difference. A Wilcoxon rank sum test was conducted for cases with persistent pain at/for 6 months postoperatively to assess if there existed any association between lingering pain at 6 months and pain scores or location at POD 0 and 2 weeks after surgery. All statistical analyses were performed by using R software (version 4.1.2).

## Results

### Patient population

A total of 112 cases of elective primary TKA were included for analysis. The average age was 65.5 ± 9.2 years, 77 (69%) patients were female, and in 64 (57%) cases, TKA was performed on the right knee. The mean BMI was 31.9 ± 4.8 kg/m^2^. The most common medical comorbidity was hypertension (50%), followed by diabetes mellitus (16%) and hyperlipidemia (14%) (Table 1).

**Table 1:** Demographics and comorbidities of 112 patients

Age^a^	65.5 ± 9.2
Female sex^b^	77 (69%)
Body mass index^a^	31.9 ± 4.8
Laterality^b^	
Right knee	64 (57%)
Left knee	48 (43%)
Comorbidities^b^	
Hypertension	56 (50%)
Hyperlipidemia	16 (14%)
Diabetes mellitus	18 (16%)
Psychiatric illness	10 (8.9%)
Gastroesophageal reflux disease	3 (2.7%)
Asthma	1 (0.9%)
Obstructive sleep apnea	10 (8.9%)
Chronic kidney disease	5 (4.5%)
History of cancer	7 (6.3%)
Venous thromboembolism	3 (2.7%)
Atrial fibrillation	3 (2.7%)
Cerebrovascular accident	1 (0.9%)
Rheumatoid arthritis	1 (0.9%)

^a^Values are given as the mean and standard deviation

^b^Values are listed as frequency (percentage of patients)

### Pain location and severity

Pain location and severity are summarized in Table 2. Regarding loss to follow-up, 100% of patients responded at POD 0, 82% at 2 weeks, 79% at 2 months, and 72% at 6 months. Overall, the most common loci of pain were patellar and medial joint line. About 3%–18% of patients who had pain were unable to pinpoint pain area (range represents different follow-up time periods). The mean pain scores by location at each follow-up time point are summarized in Supplemental Table 1.

**Table 2:** Pain locations and severity at each follow-up time point

Location	*n* (%)							
	POD 0	Average pain score	2 weeks	Average pain score	2 months	Average pain score	6 months	Average pain score
No Pain	7 (6)	0	20 (22)	0	41 (47)	0	70 (86)	0
Superior^a^	29 (28)	5.6	13 (18)	4.8	4 (9)	6.0	1 (9)	3.0
Superolateral^a^	8 (8)	5.6	2 (3)	4.0	2 (4)	4.5	0 (0)	N/A
Superomedial^a^	7 (7)	5.6	4 (6)	5.3	2 (4)	3.0	0 (0)	N/A
Inferior^a^	17 (16)	7.3	9 (13)	5.6	2 (4)	4.0	1 (9)	3.0
Inferolateral^a^	0 (0)	N/A	3 (4)	6.0	5 (11)	3.2	1 (9)	3.0
Inferomedial^a^	7 (7)	5.9	1 (1)	3.0	1 (2)	3.0	1 (9)	3.0
Medial Joint^a^	25 (24)	5.5	26 (36)	5.8	9 (19)	4.1	2 (18)	6.5
Lateral Joint^a^	12 (11)	5.4	13 (18)	5.7	4 (9)	2.8	3 (27)	3.3
Patellar^a^	51 (49)	5.9	20 (28)	6.1	15 (32)	3.9	2 (18)	1.5
Posterior^a^	17 (16)	5.4	11 (15)	4.0	8 (17)	4.1	2 (18)	7.0
Diffuse^a^	12 (11)	6.0	2 (3)	5.0	2 (4)	2.5	2 (18)	2.0

*POD* postoperative day, *N/A* not applicable

^a^Values are given as frequency (percentage of patients who reported pain)

Seven patients (6%) had no pain on POD 0. Of those who reported having pain, the most common location of pain on POD 0 was patellar area (*n* = 51, 49% of patients, mean VAS, 5.9), followed by superior (*n* = 29, 28%), and medial joint (*n* = 25, 24%). The overall mean VAS score was 5.8 ± 2.4. The highest mean VAS score was scored by those with inferior pain (7.3).

At 2 weeks postoperatively, 20 patients (22%) had no pain. Of those who reported pain, the most common location of pain at 2 weeks of follow-up was medial joint (*n* = 26, 36%, mean VAS, 5.8), followed by patellar (*n* = 20, 28% of patients), and superior and lateral joint (*n* = 13 each, 18%). The mean VAS score was 5.4 ± 2.3. The highest mean VAS score was obtained/achieved by those with patellar pain (6.1).

At 2-months postoperative mark, 41 patients (47%) had no pain. Of those who reported having pain, the most common location of pain at 2 months of follow-up was patellar (*n* = 15, 32%, mean VAS, 3.9), followed by medial joint (*n* = 9, 19%), and posterior (*n* = 8, 17%). The mean VAS score was 3.9 ± 2.2. The highest mean VAS score was scored by those with pain in superior region (6.0).

Seventy patients (86%) had no pain at 6 months postoperatively. Of those that reported having pain, the most common location of pain at 6 months of follow-up was lateral joint (*n* = 3, 27%, mean VAS, 3.3), followed by patellar, medial joint, posterior, and diffuse pain (*n* = 2 each, 18%). The mean VAS score was 3.8 ± 2.7. The highest mean VAS score was reported in those with posterior pain (7.0).

No relationship was found between having pain at the end of six months and POD 0 pain score (*P* = 0.63) or location (*P* = 0.47). No relationship was revealed between the pain at six months and pain score (*P* = 0.056) and location at two weeks (*P* = 0.88).

#### Mixed effects modeling

The time after surgery exerted a significant effect on pain score (F (3, 267.49) = 123.01, *P* ≤ 0.001, *η*_*p*_^2^ = 0.58), with the mean pain score decreasing over time (Table 3). On the contrary, the pain location had no significant effect on the pain score (F (10, 211.23) = 1.71, *P* = 0.08, *η*_*p*_^2^ = 0.07). No significant interaction was found between pain location and time point (F (25, 122.88) = 0.89, *P* = 0.62, *η*_*p*_^2^ = 0.15). Additionally, while the effect of time alone remained significant (F (3, 117.76) = 14.38, *P* ≤ 0.001), the *η*_*p*_^2^ dropped to 0.27 in the model that assessed the interaction between pain location and time point.

**Table 3:** Mean pain scores over time

Time point	Mean pain score	95% Confidence interval
POD 0	5.723	5.249–6.20
2 weeks	4.338	3.823–4.85
2 months	2.073	1.55–2.60
6 months	0.744	0.207–1.28

*POD* postoperative day

When assessing if some locations were more painful at each time point, no difference was found in pain scores between locations by Tukey’s Honest Significant Difference. However, when assessing differences in pain scores at each location over time, three locations demonstrated significant differences. In patients who reported the most serious pain at the medial joint (*n* = 47), the mean pain score was lower at two-months compared to both the day of surgery (*P* = 0.006) and two-weeks (*P* = 0.01). In patients who reported patellar pain (*n* = 66), the mean pain score was lower at six-months than on POD 0 (*P* = 0.003) and at two weeks after surgery (*P* = 0.01), but not at two-months (*P* = 0.37). The mean pain score was also lower at two-months than on the day of surgery (*P* ≤ 0.001) and at two-weeks (*P* = 0.02). There was no difference in pain score between the day of surgery and the time point of the two-weeks (*P* = 0.78). Finally, in patients who reported posterior pain (*n* = 20), the mean pain score was lower at two months than on the day of surgery (*P* = 0.026). No other time points had significantly different pain scores with the posterior location.

## Discussion

Postoperative pain remains a significant concern after TKA. In the early postoperative period, up to two-thirds of patients report pain, which can affect quality of life, patient satisfaction, and rehabilitation [7]. We sought to characterize the anatomic locations of early postoperative pain after TKA and found that over 80% of patients with pain at each time point were able to identify specific loci of pain. The proportion of patients with pain dropped and the pain subsided over time. Although the location of pain varied widely, the most common locations for knee pain following TKA were patellar (anterior), thigh (superior), and medial joint line. The most common locations of pain at 6 months postoperatively were different from those on POD 0 and at 2 weeks after operation.

Our findings in a high-volume joint replacement center showed that a focal locus of pain was identifiable and highly prevalent in the early postoperative period. Prior studies have reported anterior knee pain to be most common among patients who had pain following TKA for osteoarthritis [14–22]. However, to our knowledge, no studies examined the specific anatomic locations of pain after TKA. Knee pain mapping has been successfully used in patients (most commonly with osteoarthritis) treated non-operatively [11, 12, 14–21]. This method has been shown to be a valid and reliable technique for determining the location of knee pain, with good reproducibility [14, 16]. A recent study by Thompson et al. demonstrated that patients were not only able to identify, but also more likely to have localized pain rather than diffuse pain [14]. This study yielded similar results, as most patients were able to identify specific loci of pain, and at much higher rate than those who were unable to pinpoint their pain and reported diffuse pain. Although knee pain maps have not been utilized for post-TKA patients, the method employed in our study was able to identify specific anatomic locations of pain which were most common at early postoperative time points over a period of 6 months. Our knee pain map was developed on the basis of anatomic structures and existing validated models and may be further validated as a tool for the evaluation of post-TKA pain.

Despite our effort to characterize post-TKA pain, not a clear pattern was established with regard to location, pain intensity, and time after surgery. While we found that when looking at time alone, the time after operation accounted for 58% of the variance in pain scores. When we modeled the interaction between time and location, time after surgery only accounted for 27% of the variance in pain scores while the interaction between the two accounted for 15% of the variance. This indicates that there may exist certain relationship between time and location. Nonetheless, in this study, we failed to find any significant differences between the location with and time. It is possible that with more patients and an improved response rate, a relationship can be established. For example, as patellar and superior pain became less prominent by 2 weeks, which might be attributable to incision and tourniquet, medial pain was more pronounced. In this study, at all the time points prior to 6 months, the lateral pain was far less common than medial pain, which might be attributable to higher prevalence of medial arthritis and medial preoperative pain, as well as a more extensive medial release that is typically performed for typical varus alignment. We also found that there was no statistically significant difference in the mean pain scores between different locations, indicating that no location was statistically or clinically more painful than others, although certain locations may be more common to have pain. Moreover, patients that reported pain at medial joint, patellar, and posterior sites were found to have significantly lower pain over time. By 6 months postoperatively, there remained a low rate and even distribution of pain loci, suggesting that interventions to mitigate pain at specific anatomic locations should target more acute postoperative time frame. Additionally, while it was not found to be significant in this study, there may be some relationship between patients who report higher pain levels at two weeks and if they will have continued pain at six months, which may guide multimodal pain management strategies to be more aggressive earlier on in a patient’s post-operative course. This information may be helpful in the patient education, setting expectations, as well as further investigation on therapeutic interventions targeting these pain loci.

There are several limitations to this study. First, even though the power analysis was conducted on the basis of prior studies, the knee pain map we utilized provided 12 options for locations, leading to a spread of the reported pain locations and thus rendering an accurate a priori power analysis difficult to perform. In the future, a larger patient size should be utilized to allow for more thorough analysis of the pain locations. Second, some variations in surgical technique could not be controlled. Thirdly, only 68% of patients provided a response at all the follow-up time points. This might well raise the risk of bias in the study if the populations of those with complete responses and those with missing responses were different. This also affected the ability to assess the relationship between time and location, since this was analyzed using a repeated-measures design, which depends on having data available at each time point. Additionally, preoperative pain location and severity could not be assessed, and it is possible that patients with greater preoperative pain experienced higher-level and persisting postoperative pain. Finally, we were unable to assess whether patients provided pain responses at rest or following therapy or exercise, which might cause variations in pain experienced at the time of their response. Although TKA is performed in a relatively uniform manner, there are variations, such as implant selection, which may also influence the external validity of our study since other surgeons presumably may use different implant designs.

## Conclusions

Our study showed that in some cases, pain cause can be traced, but the majority of patients suffered from significant postoperative pain without identifiable causes. The patients were able to identify particular anatomic locations of pain, and a significant proportion of patients reported patellar, thigh, and medial knee pain in the early postoperative period. There was no relationship between pain at the end of six months and the pain score or location on POD 0 or at 2 weeks.

## Supplementary Information

**Additional file 1: Supplemental Table 1.** Mean pain scores by location at each time point.

## Data Availability

The datasets used and/or analyzed during the current study are available from the corresponding author on reasonable request. Original data in the form of articles used in this review are publicly accessible.
