# Supplementary material for: Mapping the course to recovery: a prospective study on the anatomic distribution of early postoperative pain after total knee arthroplasty
Source: Arthroplasty. 2023 Aug 3;5:37. doi: 10.1186/s42836-023-00194-3 (PMC10399043; doi:10.1186/s42836-023-00194-3)
Supplement: Supplementary file 1 — Additional file 1: Supplemental Table 1. Mean pain scores by location at each time point. [file 42836_2023_194_MOESM1_ESM.pdf]

Supplemental Table 1. Mean pain scores by location at each time point.

POD0		
Location	Mean Pain Score	95% CI
Diffuse	5.59	3.81 - 7.37
Inferior	7.44	6.06 - 8.82
Inferolateral	NA	NA
Inferomedial	5.86	3.66 - 8.05
Lateral Joint	5.56	4.01 - 7.11
Medial Joint	5.79	4.71 - 6.87
Patella	5.96	5.25 - 6.67
Posterior	6.77	5.08 - 8.46
Superior	6.44	5.37 - 7.52
Superolateral	4.06	1.80 - 6.32
Superomedial	6.61	4.61 - 8.61
2 weeks		
Location	Mean Pain Score	95% CI
Diffuse	4.94	2.09 - 7.78
Inferior	6.6	4.60 - 8.60
Inferolateral	6.39	4.06 - 8.72
Inferomedial	3.22	-0.84 - 7.28
Lateral Joint	5.56	4.30 - 6.82
Medial Joint	5.41	4.55 - 6.27
Patella	5.43	4.43 - 6.43
Posterior	4.06	2.53 - 5.58
Superior	6.02	4.22 - 7.82
Superolateral	5.72	1.69 - 9.75
Superomedial	NA	NA
2 months		
Location	Mean Pain Score	95% CI
Diffuse	1.76	-0.932 - 4.46
Inferior	4.32	1.60 - 7.03
Inferolateral	3.03	1.265 - 4.80
Inferomedial	4.2	0.359 - 8.03
Lateral Joint	2.62	0.368 - 4.87
Medial Joint	3.03	1.726 - 4.34
Patella	3.4	2.254 - 4.54
Posterior	3.47	1.89 - 5.05
Superior	5.1	3.182 - 7.01

Superolateral	4.12	0.354 - 7.89
Superomedial	1.82	-2.011 - 5.66
6 months		
Location	Mean Pain Score	95% CI
Diffuse	1.75	-0.861 - 4.36
Inferior	3.98	0.214 - 7.74
Inferolateral	NA	NA
Inferomedial	6.03	2.183 - 9.87
Lateral Joint	3.25	0.563 - 5.94
Medial Joint	3.37	-0.338 - 7.09
Patella	1.14	-1.477 - 3.76
Posterior	6.23	2.541 - 9.92
Superior	1.41	-2.329 - 5.15
Superolateral	NA	NA
Superomedial	NA	NA
